# Atlanta Medical Society

**Published:** 1892-03

**Authors:** 


### ATLANTA MEDICAL SOCIETY.

ATLANTA, GA., February 16, 1892.

President W. S. Kendrick in the chair.

Dr. Crow presented two specimens from cases, as follows :

CASE I.—Colored, age 26, from Alabama; had been suffering from severe dysmenorrhœa for two or three years, and reduced from loss of blood. Removed one ovary covered with cysts. The other being smaller cirrhotic, was not removed. Case made a good recovery and up in fourteen days. Since the operation had had some menstrual efforts—no flow of blood, but some watery discharge.

CASE II.—Age 29 years; was confined in August last. Saw her December 18th. Found her with fever, pain, chills, continuous purulent vaginal discharge, peritonitis, etc. Gave salines to prepare her, then found placental structure in uterine cavity, curetted. Also found on left side of uterus a small tumor not entirely relieved by the curetting and treatment. Operated two weeks ago and found abscess of ovary. Adhesions had to be broken up and follow broad ligament out to ovary and peal it out. Size of an orange, containing one-half pint of offensive pus. Could not tie it off, douched with ten gallons of hot water. Temperature, 99 degrees at operation. Did not get above 100 degrees after operation until eighth day, then, by a nervous excitement due to nurse leaving, it reached 103, but soon dropped down by use of sedatives and securing a nurse. Same patient since passed a renal calculi. The abscess of ovary was due to sepsis from retained placenta.

Dr. Duncan reported a number of cases of eruption, maculæ form, resembling measles, which he diagnosed erythema, temperature varying from 99 degrees to 100 degrees. Not much sick, but are sent home from school for certificate that it is not scarlet fever. One case in Surgical Institute, there for surgical treatment. He gives mild aperients, tincture rhei; if the temperature was above 103 degrees, phenacetine. There is a probability of contagiousness.

The President had seen some eight or ten cases in the last few days, and called it roseola; some enlargement of glands of neck. Gives light treatment.

Dr McRae had seen several cases, usually one in a family.

Dr. Cooper had seen several cases, with slight fever, suffusion of eyes, eruption more profuse on body, less on face. Calls it German measles, or roseola.

Dr. Crow, while away, had seen some cases which was diagnosed lymphatic fever, of epidemic form, not contagious, etc. Treats expectantly, and for sake of safety, isolates cases.

Dr. Elkin had seen several cases; called it roseola. Attacks light, and gives light treatment.

Report of case also by Dr. Elkin:

Miss P., age 24. Abscess of left kidney. Eighteen months previous health began to fail, at which time she weighed 160 lbs. She went to Texas for her health, but it failed to benefit her. After returning home she visited one of the leading gynæcologists, who dilated the cervix and urethra, with no special benefit. For months she suffered intense pains in region of kidney, with rigors and fever, which yielded to anti-malarial treatment, to return again. After some time pus appeared in urine, general health failing rapidly and losing flesh. Soon after her return, I was called to see her; found evidence of septic absorption, enlargement in region of left kidney. Putting patient under anæsthetic, cutting in, found an abscess of kidney containing about 24 ounces of pus, evacuting it and putting in drainage. Patient improved for several days, when drainage became imperfect, patient grew worse and was brought to Atlanta.

With the assistance of Drs. Cooper, Childs, Glass and Kime, I again cut in to find three pockets filled with pus, from which drainage was not good. Pus burrowing in and around kidney, opened up all pockets, putting in three large drainage tubes and packed wound with iodoform gauze, which was kept up until death of patient from septicæmia. Kidney (post-mortem) was found all honeycombed by destructive processes, scrapings from which did not reveal any special evidence of tuberculosis microscopically, although patient had two aunts to die of tuberculosis, and the patient had suffered from glandular enlargement. Nothing short of nephrectomy would have relieved case. Her condition after first operation would not warrant such a procedure.

At time of first operation, there had been severe pain in region of the other kidney, and fearing it would become implicated, it was desired to save as much of left kidney as possible, hence nephrectomy was not performed at the time.

Dr. Crow.—I had a similar case, whose father and mother died of tuberculosis. Dr. D. H. Agnew diagnosed trouble with kidney and sent her South. First saw her with temp. 103 deg.; found pus with aspirator, cut down next morning, giving free drainage, washing out with hydrogen peroxide one half water, using it every day for two weeks, discontinued on account of irritation, then used listerine. Patient recovered. Had cystitis; could inflate bladder from incision. The Reference Hand-book of Medicine gives 72 cases of recovery, mine making the 73d. I think free drainage the cause of recovery.

Dr. Hardon reported a case of ovarian cyst, in a woman 58 years of age, 6 years in developing. Resembled a woman 9 months pregnant, some symptoms due to pressure. Operated, drew off most of fluid. Adherent over whole of anterior surface; separated by use of fingers, without tying any points. Posterior surface not adherent. Found a cyst in back upper part, which contained a colloid material, which could not be evacuated, being jelly like; flushed out abdomen, used rubber drainage tube. Had a good pedicle. Patient recovering nicely except cannot evacuate bladder, due to lax condition of bowel wall, and want of tonicity of parts.

Dr. Cooper.—The nearest approach to Dr. Elkin's case that I have had, was a case of peri-nephritic abscess in a woman. No tumor. Thigh flexed, characteristic of such conditions. Aspirated second time before finding pus, then cut down giving drainage. Case report: Tumor in ischio-rectal fossa—rare location for a tumor—existed 15 years. Recognized by patient as a hard tumor. It was against wall of rectum. With one finger in rectum, dissected it out, leaving only the thin rectal wall, brought the tissues together by cat gut sutures, one row above another, completely closing the cavity and wound, getting union by first intention. Diagnosed as sebacious cyst, found correct on incising it.

Second case. Urethrotomy. Man of 42 years, had stricture for 7 years. Very weak, anæmic, with cystitis, kidney and lung complication. Operated on 3 years previously by the late Dr. Westmoreland. Good effect of operation kept up for sometime, by passage of steel sound, but neglected it and fell into the hands of a homœopath, who treated him with medicines, claiming that he could cure him without operation. Cavity in one lung. Urine 10 to 50 per cent. albumen by volume, chronic crystitis. Tried one month to pass filliform. At last succeeding, operated. From coughing and straining, abscess of lung bursted and came near strangulating patient. Cut down on filliform, through dense cicatricial tissue. No bleeding scarcely. Stitched in full sized catheter for drainage, washing out each day. Left catheter in for 8 1-2 days. Patient did well and had no urethal fever.

Dr. McRae reported a case of convulsions. Woman pregnant. Had one convulsion six weeks previous. Gave chloroform, veratrum viride, etc. Six weeks after first convulsion, they returned. Performed podalic version. Os contracted in mouth of child, retaining it fifteen minutes, although patient was under influence of chloroform. Patient has done well since, and no fever. Dr. McRae also reported one case of sebacious cyt removed from under tongue. Second case in girl 16 years old. Had been developing since she was 4 years old. I did seven urethrotomies last year, and used perineal drainage in them. I believe I was the first to use perineal drainage in Atlanta. One case I operated, on had to do it without guide. Urethra impervious. Find excellent results from the perineal drainage. Use a safety pin and antiseptic dressing and convey discharge to vessel by side of bed. In most of the cases there were fistulous communications.

